# Staff Experiences Delivering Hospital Outreach Post‐Suicidal Engagement (HOPE) in a Regional Context: A Qualitative Study

**DOI:** 10.1111/inm.70401

**Published:** 2026-11-10

**Authors:** Corina Modderman, Maria Veresova, Rachael McAleer, Lisa Hanson, Laura Hemming, Phil Maude, Evelien Spelten

**Affiliations:** ^1^ Violet Vines Marshman Centre for Rural Health Research, La Trobe Rural Health School La Trobe University Bendigo Campus Bendigo Victoria Australia

**Keywords:** aftercare, mental health nursing, multidisciplinary workforce, post‐suicidal care, regional health, suicide prevention

## Abstract

Suicide rates remain disproportionately high in regional Australia, where geographical, workforce and service‐system constraints can limit access to post‐suicidal care. This study explored staff experiences of delivering the Hospital Outreach Post‐Suicidal Engagement (HOPE) program across three regional hospital sites in Victoria, Australia. Semi‐structured individual interviews and one focus group were conducted with 16 HOPE staff, including nurses, peer workers, psychosocial support staff and allied health professionals. Data were analysed using reflexive thematic analysis, with reporting guided by the COnsolidated criteria for REporting Qualitative research (COREQ) checklist. Four themes were identified: regional influences on HOPE service delivery; adaptations to individual consumer needs; workforce capacity and teamwork; and HOPE's integration with the broader healthcare system. Staff valued HOPE's multidisciplinary and person‐centred approach but identified barriers associated with geographical distance, infrastructure, workforce capacity and fragmented service pathways. The findings demonstrate how regional context shapes the delivery of post‐suicidal care and highlight the importance of adequately resourced, flexible and integrated service models. These findings have implications for multidisciplinary HOPE practice, workforce planning and continuity of post‐suicidal care across regional communities.

## 1 Introduction

Suicide remains a major public health concern globally and in Australia, where 3214 suicide deaths were recorded in 2023, representing a rate of 11.8 per 100 000 people (Australian Institute of Health and Welfare 2026). Suicide rates are higher in many regional, rural and remote communities than in metropolitan areas (Australian Institute of Health and Welfare 2026). In this study, the term ‘regional’ is used to describe non‐metropolitan locations (Department of Health 2026).

People living in regional communities may experience reduced access to mental health services, longer travel distances, workforce shortages, stigma, social isolation and increased availability of lethal means (Casant and Helbich 2022; Houston 2024; Kavanagh et al. 2023). These challenges can affect both the immediate crisis responses and continuity of care following a suicidal crisis. Addressing suicide therefore requires approaches that respond to the psychological, social and structural factors influencing a person's wellbeing (Modderman, Veresova, McAleer, et al. 2026b).

A previous suicide attempt is one of the strongest predictors of future suicidal behaviour (Beghi et al. 2013), highlighting the importance of post‐suicidal care. Evidence suggests that effective post‐suicidal care services are integrated with broader health and community service systems, commence engagement as early as possible, and provide flexible, assertive and clinically supported follow‐up that is informed by the perspectives of people with lived experience. Although lived‐experience involvement is increasingly recognised as important in suicide prevention and post‐crisis support, research examining how it is actively incorporated into these services remains limited (Martin et al. 2023; Purdon et al. 2025).

Despite the importance of post‐suicidal care, less is known about how these services are experienced by staff in regional settings. Regional service delivery may be shaped by geographical distance, local workforce capacity, infrastructure, referral pathways and the availability of complementary services. Therefore, further research is needed to understand the contextual and organisational factors that enable or constrain the delivery of post‐suicidal care in regional communities.

## 2 Background

The Hospital Outreach Post‐Suicidal Engagement (HOPE) program was established in Victoria, Australia, with funding from the Victorian Government as part of the Suicide Prevention Framework 2016–2025 (Department of Health 2016). HOPE is a community‐based post‐suicidal care service that provides intensive, short‐term support for up to 12 weeks following a suicidal crisis. Multidisciplinary HOPE teams include mental health professionals and peer support workers with lived experience. They work collaboratively with consumers to deliver flexible, tailored, person‐centred support (Better Place Australia 2025).

The HOPE model is designed to address the complex and interconnected needs that may follow a suicidal crisis. Support may include psychological and psychiatric care, housing assistance, financial counselling, substance‐use services, family‐violence support, employment assistance and cultural services (Better Place Australia 2025).

HOPE has been widely implemented across Victoria, and a growing body of research has examined its outcomes (Kehoe et al. 2023; Williamson et al. 2021; Wright et al. 2021; Innes et al. 2026). Studies involving adults have reported reductions in suicidal ideation and psychological distress, as well as improvements in coping self‐efficacy, hopeful thinking and overall wellbeing (Kehoe et al. 2023; Williamson et al. 2021; Wright et al. 2021; Innes et al. 2026). Young people who have accessed HOPE have also reported reduced suicide risk and increased quality of life (Meyer et al. 2024), while parents have described the program positively (Webb et al. 2025). HOPE engagement has additionally been associated with reduced reliance on emergency departments and other hospital services (Wright et al. 2021).

Existing research indicates that the characteristics and practices of HOPE staff are central to the delivery of the program (Kehoe et al. 2023; Williamson et al. 2021; Wright et al. 2021; Innes et al. 2026). However, previous research has focused primarily on consumer, carer and service outcomes, with limited attention to staff experiences of delivering HOPE. In addition, existing evaluations have largely been conducted in metropolitan settings (Kehoe et al. 2023; Williamson et al. 2021; Wright et al. 2021; Innes et al. 2026). These findings may not fully reflect the geographical, workforce, infrastructure and service‐system conditions that shape post‐suicidal care in regional communities. Consequently, little is known about how regional context and organisational conditions shape the delivery of HOPE.

This study aimed to explore staff experiences of delivering the HOPE program across three regional hospital sites in Victoria, Australia. Specifically, it examined how geographical, organisational, workforce and service‐system factors shaped the delivery of responsive, person‐centred and continuous post‐suicidal care. The study also explored how collaboration between HOPE teams and other health and community services enabled or constrained care delivery in regional settings. The research question was: What are the experiences of staff delivering the HOPE program in regional settings?

## 3 Methods

### 3.1 Study Design

A qualitative descriptive design was used to explore staff experiences of delivering the HOPE program across three regional hospital sites in Victoria, Australia. This design was appropriate because the study aimed to produce a clear, practise‐oriented account of staff experiences and of the organisational, geographical, workforce and service‐system conditions that shaped care delivery. Such an account can inform service planning by showing how these conditions affect the delivery of post‐suicidal care in practice. Qualitative description also supported analysis that remained close to participants' language, while recognising that the findings reflect interpretation within the social, organisational and temporal context in which the data were generated (Kim et al. 2017; Furidha 2023).

Reporting was guided by the COnsolidated criteria for REporting Qualitative research (COREQ) checklist (Tong et al. 2007). Ethics approval was granted by the La Trobe University Human Ethics Committee (HEC 25032).

### 3.2 Participant Selection and Recruitment

Staff members working in the HOPE program at the three participating regional hospital sites were eligible to participate. A total population sampling approach was used, whereby all eligible staff across the three sites were invited to participate rather than a purposive subset. Site managers distributed an email invitation and participant information sheet to all eligible staff. Site managers' role was limited to distributing the study information; participation was voluntary, and staff were not required to inform their manager whether they participated. Because the invitations were distributed by site managers, the researchers could not determine the total number of eligible staff who received the invitation or declined participation.

None of the authors hold managerial or supervisory responsibility for participants, and participation or non‐participation had no effect on participants' employment or relationship with the HOPE program. Participants received a gift card in acknowledgement of their time and contribution; this was explained in the participant information sheet.

A demographic questionnaire collected information about participants' characteristics, including their employment site. Employment site was not reported because the small number of participants at each site meant that combining site with professional role and other characteristics could increase the risk of identification.

### 3.3 Procedure

Interested staff provided informed consent through an online survey and supplied an email address for study contact. Following consent, the second author contacted participants to arrange a mutually convenient time for data collection. Participants could choose between attending a pre‐scheduled focus group or completing an individual interview. Offering both formats was a pragmatic decision intended to support accessibility and convenience for staff working across geographically dispersed sites. The formats were used to elicit staff accounts, rather than to generate different types of data or to compare the formats. The same core topics were explored in both. Staff who did not attend the focus group were offered an individual interview to minimise exclusion because of scheduling or availability.

All sessions were conducted online using Microsoft Teams. The second author coordinated and scheduled the sessions directly with participants. All authors were involved in individual interviews and/or facilitated the focus group. With participants' consent, all sessions were audio‐recorded and transcribed for analysis.

### 3.4 Data Collection

Data were generated through 12 semi‐structured individual interviews and one focus group involving four participants. All sessions were conducted online using Microsoft Teams. The second author coordinated the sessions and scheduled them directly with participants after consent had been obtained. All authors were involved in individual interviews and/or facilitated the focus group. With participants' consent, all sessions were audio‐recorded and transcribed for analysis.

### 3.5 Interview and Focus Group Schedule

The interview and focus group topic guide was developed by the research team to address the study aim and research question. It was informed by the researchers' experience in suicide prevention and rural health systems (Modderman and Sanders 2026; Modderman, Veresova, Canosa, et al. 2026; Modderman, Veresova, McAleer, et al. 2026a) and HOPE service delivery (Better Place Australia 2025; Kehoe et al. 2023; Williamson et al. 2021; Wright et al. 2021; Innes et al. 2026). It consisted of open‐ended questions exploring five broad areas: challenges, enablers, collaboration, suggested improvements to the HOPE model, and additional resources or strategies that could strengthen care delivery. Follow‐up prompts were used where appropriate to clarify participants' responses and encourage further examples. The same core topics were explored in the individual interviews and focus group, while allowing the discussion to develop according to participants' experiences. The full topic guide is provided in Supplementary File 1.

### 3.6 Data Analysis

Data were analysed using reflexive thematic analysis, informed by Braun and Clarke's six‐phase approach (Braun and Clarke 2006, 2014). This approach was selected because it supports the development of interpretive themes from patterns of meaning across qualitative data while recognising the active role of researchers in producing the analysis. Themes were therefore understood as interpretive outputs developed through engagement between the researchers, the dataset and the study context, rather than as categories existing independently within the data (Braun and Clarke 2021).

The six phases involved familiarisation with the data, generating initial codes, searching for themes, reviewing themes, defining and naming themes and producing the analytic account (Braun and Clarke 2006, 2014). The first three authors familiarised themselves with the interview and focus group transcripts and recordings. They independently coded an initial subset of the transcripts, including material from both the individual interviews and the focus group. Coding was primarily inductive, grounded in participants' accounts, while being informed by the researchers' existing knowledge of regional health service delivery and post‐suicidal care (Modderman and Sanders 2026; Modderman, Veresova, Canosa, et al. 2026; Modderman, Veresova, McAleer, et al. 2026a). The researchers compared and discussed their interpretations, and codes were revised, combined, separated or discarded as the analysis developed. They then coded the remaining transcripts and continued to discuss emerging codes and potential themes during research meetings with all authors. These discussions supported reflexive consideration of similarities, differences and alternative interpretations; they were not undertaken to establish statistical inter‐coder reliability.

Codes were subsequently grouped into potential themes, which were reviewed against the coded extracts and the dataset as a whole. Themes were refined, defined and named before being organised into a coherent analytic account. The final themes were developed across the combined interview and focus group dataset and were supported with illustrative participant quotations (Braun and Clarke 2006). NVivo software (Lumivero 2025) was used to store the transcripts, organise codes and facilitate retrieval of coded data. All authors contributed to discussion of the developing thematic interpretation.

### 3.7 Reflexivity and Positionality

The research team included six women and one man with backgrounds in psychology, social work, nursing and physiotherapy, as well as experience in suicide prevention and qualitative health research. The researchers recognised that their professional training, practice experience and perspective on regional health service delivery could influence the research questions, interactions with participants and interpretation of the data. Reflexive discussions were undertaken throughout the analysis to consider how the researchers' backgrounds, experiences and assumptions may have influenced coding and theme development (Braun and Clarke 2014). Differences in interpretation were discussed to identify alternative readings of the data and to support a considered, transparent analytic process.

## 4 Findings

Sixteen HOPE staff participated in the study. Twelve individual interviews and one focus group (*n* = 4) were conducted, with each session lasting approximately 1 hour. Most participants were women (*n* = 13) and participants ranged in age from 25 to 64 years. The participant group included nurses (*n* = 7), psychosocial support workers (*n* = 3), peer/lived‐experience workers (*n* = 2), support staff (*n* = 1), social workers (*n* = 1), psychologists (*n* = 1) and mental health workers (*n* = 1). Four participants had worked in HOPE for less than 1 year, eight had worked in HOPE for 1–5 years, and four had worked in HOPE for more than 6 years. Participant characteristics are presented in Table 1, which reflects participants from both the individual interviews and the focus group.

**TABLE 1:** Demographics.

Characteristic	Category	*n*
Gender	Female	13
	Male	3
Age	25–34 years	4
	35–44 years	3
	45–54 years	5
	55–64 years	4
Current role	Nurse	7
	Psychosocial support worker	3
	Peer/lived experience worker	2
	Support staff	1
	Social worker	1
	Psychologist	1
	Mental health worker	1
Years working in the HOPE program	Less than 1 year	4
	1–5 years	8
	More than 6 years	4

*Note:* Employment site was collected as part of the demographic questionnaire but is not reported. Because of the small sample size, there is potential for participants to be identifiable when site is considered alongside role and other demographic characteristics.

Four themes were identified in the data. The first examines how regional conditions and features of the HOPE model shaped service delivery. The second describes how staff adapted care to respond to individual consumer needs. The third focuses on workforce capacity, teamwork and staffing arrangements within HOPE. The fourth explores relationships between HOPE and the broader healthcare system, including referral pathways, collaboration and access to complementary services. Although these themes are distinct, they are interconnected in the delivery of post‐suicidal care in regional settings.

### 4.1 Theme 1: Regional Conditions and the HOPE Model of Care

Participants described how the regional environment, available resources and operational features of the HOPE model shaped the delivery of care. These conditions could support engagement but could also limit privacy, accessibility, continuity and the program's capacity to respond to consumer need. As part of a larger hospital network, HOPE enabled collaboration and resource sharing. One HOPE office was located in a building that was not exclusively used for healthcare services, which staff thought may have benefited consumers:

People who have never accessed [mental health] services before might feel more comfortable coming to a space that isn't specifically a mental health service. (Participant 7)

However, the shared building was often crowded and noisy, making it difficult to find quiet and private consultation rooms. One participant explained:

We've got really vulnerable people we're trying to have conversations with. And sometimes, you know, really sensitive topics. People with all sorts of sensory issues and stuff as well. And it is really challenging for us to find somewhere sometimes that is going to be an appropriate place that they can feel really comfortable and not at least have some sort of noise. (Participant 5)

Participants suggested making the space more inviting and sensory‐friendly via ‘dim lights. […] Something that's not super bright like a dentist office’ or ‘comfortable chairs’ (Participant 15).

Large regional catchment areas also affected the practical delivery of outreach. Travel to face‐to‐face appointments was time‐consuming, and staff often drove long distances to visit consumers who could not travel to the office:

You end up spending two hours travelling. An hour and a half appointment. Two hours back. There's your whole day gone. (Participant 14)

This issue was further complicated by insufficient vehicles. At one of the HOPE sites, it was reported that there was only one car shared between all team members, which ‘does impede the service’ (Participant 14). Additional vehicles were viewed as necessary to support more efficient outreach for people without access to public transport.

Some participants reported that staff often pay out of pocket for small therapeutic supplies, such as journals or fidget toys, due to a lack of funding. More discretionary funding for HOPE means staff would ‘be able to not spend [their] own money on things’ (Participant 11).

HOPE predominantly only operated during business hours. Participants noted that ‘the majority of [consumers'] acute distress and crisis happens outside of business hours’ (Participant 7). Although round‐the‐clock crisis services were available, consumers could not access their usual HOPE team during these periods, despite having already established rapport with those staff.

Participants also identified eligibility requirements as a feature of the HOPE model that could restrict access to care. HOPE excluded some consumers with co‐occurring conditions, including acute substance use or complex psychiatric diagnoses. Some participants questioned whether this was appropriate:

What's that saying, there should be no closed door. But substance use is often seen as someone else's problem and not [emergency departments] or mental health services. They get stuck with a need and nowhere to go. (Participant 13)

Some participants wanted the eligibility criteria broadened to accommodate a wider range of consumers. However, they recognised that broader eligibility would increase referrals to a service that already had insufficient staff to meet demand. Overall, participants described a service shaped by the realities of regional delivery. Shared facilities and links with the wider hospital network created some opportunities for access and collaboration, while distance, limited resources, restricted operating hours and eligibility requirements affected how consistently HOPE could respond to consumer needs.

### 4.2 Theme 2: Flexible and Individualised Care

Participants valued HOPE's holistic and multidisciplinary approach and described flexibility as central to providing care that responded to individual consumer needs. This flexibility included the type, location and timing of support, as well as adapting communication and assessment processes. Participants emphasised that HOPE provided therapeutic interventions, medication, financial assistance, social support and support for families and carers:

You come to HOPE and you can receive all of those services and we do that really well as a group, which I think is really, really important (Participant 8)

HOPE team members discussed targeted support according to consumers' needs, including flexibility in the type of support provided and the time or location of appointments:

If somebody doesn't like coming into [the office], we can meet them at their house, we can go for a walk. We try to make it as comfortable as possible for the people that we're working with (Participant 11).

Participants also identified the importance of hearing directly from consumers about their experiences of the program. They suggested that HOPE should be shaped by the voices of the people it was designed to support. However, no routine process was in place for staff to receive and analyse consumer feedback. One participant suggested using comments included in discharge paperwork:

At the end of our [paperwork] we had to cover a little sphere about ‘How did the HOPE program help you?’ or something or ‘Can you give some feedback about what made things different?’ or something like that. […] That's on that but we don't keep that anyway because it gets scanned into our system. So, from our end we could probably do some qualitative stuff around comments (Participant 6)

Flexibility was also influenced by the accessibility of communication and assessment processes. Some participants described difficulties using interpreter services in regional areas because interpreters could be members of the consumer's community:

The interpreters are people from [the consumer's] community, or [consumer] can't verify that they're not from their community, so they feel unwilling to engage with you in that space (Participant 2)

Participants noted that these concerns could affect engagement because of confidentiality concerns and stigma surrounding suicide in some communities.

While HOPE was generally considered well‐tailored to individual needs, participants suggested that assessment materials and procedures could be improved for neurodivergent consumers. For example, they suggested reducing the length of questionnaires:

It's a double‐sided page and it can be quite overwhelming for somebody who's neurodiverse to see that and take that in and think, I now need to–I'm already feeling quite miserable, now I need to sit here and try and navigate my way through this as well (Participant 2)

Participants portrayed HOPE as a service that could be adapted around the individual circumstances of consumers. Support was provided in different forms and settings, with staff also identifying the need for more systematic consumer feedback and more accessible communication and assessment processes.

### 4.3 Theme 3: Workforce Capacity and Teamwork

Participants described teamwork and multidisciplinary practice as important strengths of HOPE. At the same time, staffing levels, caseload capacity, supervision and access to specialised roles affected the team's ability to provide flexible and responsive care. Some participants described limiting caseloads as helpful because reasonable caseloads gave staff greater capacity to provide additional support when required:

If you need to sit with a consumer for a little bit longer than what the allocated session time was, you're able to have that flexibility at times, which is really good (Participant 14)

Positive sentiments about fellow HOPE team members and leadership were expressed by most participants:

Our team is kind of like a hidden gem in a service, that's what it feels like (Participant 15)

Most HOPE team members described working well together. Discussing consumer care as a team enabled staff to share knowledge and experience:

We will speak to each other about our clients and what's happening with them, and what other people suggest. And that teamwork, I think, is really important because we're all at different levels and we've got different qualifications and it's good to be able to talk to other people. [A team member] might have had a client with similar events that have happened, and they might have some experience about what happened with that person that could be helpful for you (Participant 11)

Most participants expressed that they wanted opportunities for ‘more formalised training every now and again just to update skills’ (Participant 11), and for formal supervision outside their immediate team. Participants also suggested that additional specialised roles could strengthen the program, including family/carer peer workers, outreach workers and referral or intake workers:

One of the things in our model of care that we'd probably like to do is to have maybe a more regular system of assertively looking for people in our service who might meet criteria for our program. If we had someone who was solely a clinician dedicated to, I don't know, being a referral and intake worker, it would mean that that one person, that's their job (Participant 7)

However, participants identified difficulties recruiting to specialised positions. One participant described repeated unsuccessful attempts to recruit a family/carer peer worker:

One of the things that we had factored in the budget was a family carer peer worker, but we've not been able to recruit to the position. So here we are sort of nearly two years down the track and we've had three rounds of advertising for that position and haven't been able to recruit into it. I think that there's a couple of reasons for that. […] [One of them is] a regional factor (Participant 3)

Participants also linked workforce sustainability with appropriate wages and recognition across professional and lived‐experience roles. One participant described concerns about pay differences within mental health and between mental health and general nursing:

I think always, and this will probably be an issue forever, but appropriate wages and recognition for everyone. There's still a huge pay gap in mental health. Also comparing mental health to the general workforce, I think at the moment there's around a $20,000 pay gap between mental health nurses and mental health social workers, the social workers being on the low end of that stick. Also, being acutely aware that our lived experience and peer support and wellbeing workforce are probably not paid anywhere near enough. Then, the point that mental health nurses are paid less than general nurses, there's a very real concern at the moment that particularly allied health staff are going to leave the workforce because they're not paid anywhere near close to their colleagues that are doing the same job. It can be hard to look after people when we have our own money concerns and stuff like that (Participant 8)

Participant 11 observed that ‘there's just enough staff’ to meet consumer demand. Unexpected staff absences placed additional pressure on the remaining team members and ‘makes it all a bit hard to deliver the service properly’ (Participant 11).

Overall, most participants described a committed and collaborative workforce that supported shared decision‐making and flexible care. They also reported that limited staffing, recruitment difficulties, gaps in training and supervision, and concerns about pay and recognition placed pressure on the team's capacity.

### 4.4 Theme 4: HOPE Within the Broader Healthcare System

Participants described HOPE as dependent on relationships with other health and community services. Collaboration supported referrals, but fragmented pathways and limited capacity in other services constrained the program's reach. Most participants reported strong collaborative relationships with other regional services. They attributed this partly to HOPE staff proactively building relationships:

[Our] staff will go around the [area] and we do a lot of networking and education sessions with services such as general practitioners and other service providers as well, to talk about our HOPE program and what we do, how you refer, so that everyone's aware of our service (Participant 8)

HOPE staff regularly attended multidisciplinary team meetings with other services. This helped teams remain connected to former consumers and informed about potential new referrals:

We get invited to multidisciplinary team meetings with those services if they have somebody that they feel might be appropriate for referral, or if they're supporting someone that's previously been in our program that we've stepped up (Participant 2)

However, collaboration was not always seamless. Some services remained unaware of HOPE despite the team's outreach, meaning that eligible consumers could be missed:

We notice that [staff in other services] are not sending people to HOPE. You have to do a lot of liaison with them and there is not always the time to do that or follow through. So people fall through the cracks (Participant 13)

At other times, services knew about HOPE but were unclear about its eligibility criteria:

They're like, ‘Oh, we should just refer to [HOPE]’ but not knowing the criteria and everything. But they've already made this plan with the person. And that really puts us in a tricky spot because then we declined them. And then all this becomes a whole problem (Participant 15)

Participants suggested that HOPE teams provide more routine information sessions to clarify eligibility criteria, referral pathways and alternative services:

What the appropriate pathways are, what the alternative options are, that people could go to short‐term for a week or two for some stabilisation and crisis support, prior to coming to us (Participant 2)

Participants also described broader shortages in the healthcare system. Long waiting times for accessible and affordable mental health services made continuity and transitions between services difficult:

If we could have access to bulk‐billing counselling and psychology with no out‐of‐pocket it would just… Oh, it would just be life changing for so many people that could not otherwise have that support (Participant 5)

Community services were also described as insufficiently funded and resourced. Participant 13 stated that ‘there is a drug and alcohol service here, but it is never enough’. The same participant added that ‘community mental health could offer a lot of what HOPE provide if they had the resources and staffing to do so’.

HOPE was described as closely connected to the wider regional health and community service system. Relationships and regular contact supported referrals, although uneven knowledge of the program, unclear referral pathways and shortages across other services made it difficult for some consumers to access appropriate ongoing support.

## 5 Discussion

Staff delivering HOPE across three regional Victorian sites described a program shaped as much by its regional setting as by its formal model of care. Four themes captured these experiences: regional conditions and the HOPE model; flexible and individualised care; workforce capacity and teamwork; and HOPE's position within the broader healthcare system. Together, these themes extend existing HOPE research (Kehoe et al. 2023; Williamson et al. 2021; Wright et al. 2021; Innes et al. 2026) by demonstrating how regional conditions influence the delivery of post‐suicidal care. Consistent with broader research on mental health access in rural Australia, staff identified distance and transport as constraints on care (Kavanagh et al. 2023). The findings also provide a service‐level account of how regional conditions shaped care beyond whether consumers could reach a service: staff described limited access to private and suitable consultation spaces, interpreters and vehicles, as well as service hours that did not always align with periods of acute distress. This shows how regional context can affect the conditions and continuity of care within a post‐suicidal service. In these settings, the delivery of the model depended not only on its clinical components but also on the availability of suitable spaces, transport, interpreters, referral pathways and a sufficiently resourced workforce (Kavanagh et al. 2023).

Participants reported the regional environment as both enabling and constraining. Locating HOPE within the broader hospital network, rather than in a clearly identifiable mental health facility, was viewed as potentially supporting first contact for consumers. However, shared hospital spaces may also compromise privacy and the conditions needed for sensitive conversations. Staff described conducting sensitive conversations in rooms that were poorly suited to consumers with sensory sensitivities. Long travel distances, limited access to vehicles and service hours that did not always correspond with periods of acute distress further constrained care delivery. These findings demonstrate how regional service infrastructure can shape mental health care, challenges also identified in the broader rural mental health literature (Houston 2024; Kavanagh et al. 2023).

HOPE's eligibility criteria generated some concerns among participants. Staff reported that consumers with substance use or complex psychiatric presentations were sometimes excluded, despite being similarly ineligible for other services. In a regional context where alternative services are limited (Bloch‐Atefi et al. 2021), exclusion from HOPE may leave consumers without a clear pathway for support. At the same time, participants identified the flexibility of HOPE's intervention as a strength. Medication assistance, financial support and family involvement were described as components of an integrated response rather than as discrete services requiring referral elsewhere. Staff also adapted the location of care, meeting consumers in their homes or other community settings when office‐based appointments were unsuitable. These accounts align with rural mental health literature emphasising the importance of service models that respond to local circumstances and develop integrated care through relationships between providers (Fitzpatrick et al. 2017). They also reflect the multidisciplinary combination of clinical and psychosocial support described in evaluations of post‐suicidal aftercare and HOPE (Kehoe et al. 2023; Williamson et al. 2021; Wright et al. 2021; Innes et al. 2026). This study adds detail about how staff enacted that flexibility in practice: they brought different forms of support together and adapted where care took place in response to consumers' circumstances. The findings suggest that HOPE's multidisciplinary structure enabled staff to exercise professional judgement and adapt care to individual and regional circumstances, rather than applying a single, standardised mode of delivery.

However, participants reported that feedback from consumers was not routinely collected or used to improve the service. Although a comment section was included in discharge documentation, the feedback was not retained or analysed. Establishing a consistent process for collecting and reviewing consumer feedback may help ensure that consumers' experiences inform service planning and evaluation (Purdon et al. 2025). Assessment processes were also described as poorly suited to neurodivergent consumers, with lengthy standardised forms experienced as overwhelming during periods of distress. This concern is consistent with wider critiques of mental health assessment tools that have not yet been designed with neurodiverse individuals in mind (Goodwin et al. 2025; Moore et al. 2024).

Workforce capacity was another important condition of care. Reasonable caseloads enabled staff to extend appointments when required. Participants also valued the team's multidisciplinary composition, describing how colleagues drew on different professional qualifications and prior experiences. One participant raised concerns about remuneration and recognition across professional and lived experience roles. These accounts reflect staff perceptions rather than an independent assessment of remuneration; however, in regional services, where recruitment and retention are already challenging (Kavanagh et al. 2023; Fitzpatrick et al. 2017), remuneration and recognition may be especially important to sustaining the workforce required.

HOPE's connection with the wider healthcare system depended on relationships developed by individual staff. Networking and participation in multidisciplinary meetings helped staff explain the service and maintain its visibility. This reliance on relationships is consistent with rural mental health research, which highlights trust, local knowledge and responsiveness to local needs as important to developing sustainable service linkages (Fitzpatrick et al. 2017). These relationships were nevertheless fragile, and participants reported that some services did not refer to HOPE despite this outreach. This echoes Australian research on rural mental health referrals, which describes varied referral processes and challenges with service coordination and navigation (Bartel et al. 2025). The present findings show how that system challenges played out for HOPE: limited awareness and uncertainty about eligibility could mean that potential referrals were missed, while staff had to devote time to outreach. Participants identified improved access to low‐cost psychology and counselling as a substantial unmet need, while local alcohol and other drug services were described as chronically under‐resourced relative to demand. These concerns align with broader evidence that cost and limited service availability can restrict access to mental health care in regional, rural and remote Australia (Kavanagh et al. 2023; Bartel et al. 2025). HOPE therefore operated within a broader service system whose capacity directly influenced the options available to consumers after referral.

Findings suggest that HOPE in regional settings is best understood as a context‐dependent and relational model of post‐suicidal care. The program's value lies in its capacity to provide flexible, multidisciplinary and individualised support; however, this capacity depends on conditions beyond the program itself. Privacy, transport, eligibility pathways, interpreter access, assessment processes, workforce stability and relationships with other services all shaped what HOPE could provide in practice. Regional post‐suicidal care therefore cannot be strengthened through program‐level changes alone. It also requires investment in the wider health and social care infrastructure on which the program depends.

These findings have practical implications for service planning. HOPE services should have access to suitable private consultation spaces, adequate transport and staffing, culturally safe interpreting options and regular professional development. This could include reliable access to private rooms for sensitive conversations, transport arrangements that account for travel time between communities and interpreting options that can be arranged promptly when required. Assessment processes could be reviewed with neurodivergent consumers and staff to identify ways to make forms and appointments more manageable during distress. Referral pathways should be supported through ongoing communication with health and community services, including clear information about eligibility and alternative options for consumers who cannot be accepted into HOPE. Regular information sessions with referring services, accompanied by a concise guide to HOPE eligibility and local alternatives, could help reduce uncertainty and missed referrals. Where a person is ineligible, an agreed process for linking them with another service could reduce the risk of them being left without a clear support pathway. Routine collection and review of consumer feedback could further support service improvement and ensure that the model remains responsive to the people it is intended to support. For that feedback to inform change, it should be retained and reviewed regularly by the service. Future research should examine how models such as HOPE can be adapted to support accessibility and continuity of care in regional settings.

### 5.1 Strengths and Limitations

This study has several strengths. To the authors' knowledge, it is the first published study to examine staff experiences of delivering HOPE in regional settings across three Victorian sites. It also contributes staff perspectives to the HOPE literature and identifies practical considerations relating to program delivery.

Several limitations should be considered. Recruitment was facilitated by site leaders, who distributed the study invitation to eligible staff. Because the total number of eligible staff who received the invitation was unknown, a response rate could not be calculated. The voluntary recruitment process may have resulted in self‐selection, as staff with particularly positive or negative views may have been more likely to participate.

The study was conducted across three regional Victorian HOPE sites and relied on participants' accounts of their experiences. The findings may therefore not be transferable to services with different organisational structures, staffing arrangements, cultural contexts or geographical settings. The perspectives of consumers, carers, program administrators and collaborating services were not included. In addition, participants chose either an individual interview or a focus group, meaning that the data were generated in different interactional contexts. The focus group may have been influenced by group dynamics and social desirability, while individual interviews allowed more private exploration of participants' experiences.

The study describes staff experiences of HOPE delivery but does not directly measure consumer outcomes, such as changes in suicidal behaviour, wellbeing or service engagement.

## 6 Conclusion

This study shows that the delivery of HOPE in regional Victoria is shaped by the interaction between the program and the wider service environment. Staff valued HOPE's flexible, multidisciplinary and person‐centred approach, but described how geographical distance, limited transport, workforce constraints, restrictive eligibility criteria and fragmented referral pathways limited what the program could provide.

The key implication is that improving regional post‐suicidal care requires both a responsive model of care and the infrastructure needed to sustain it. Future research should incorporate consumer and carer perspectives and examine service and consumer outcomes across different regional settings. This would provide a more comprehensive understanding of how post‐suicidal care can be made accessible and responsive in regional communities.

## 7 Relevance for Clinical Practice

HOPE staff play an important role in delivering and coordinating post‐suicidal care in regional settings. This study highlights the value of flexible, person‐centred practice, multidisciplinary teamwork and strong relationships across services. It also demonstrates how geographical distance, workforce shortages, limited resources and fragmented referral pathways can constrain continuity of care. These findings are relevant to professionals working across multidisciplinary HOPE teams. Adequate staffing, clinical supervision, professional development and integrated service pathways may strengthen the accessibility and sustainability of post‐suicidal care in regional communities.

## Funding

This work was supported by the Murray Primary Health Network.

## Ethics Statement

Full ethics approval was obtained from La Trobe University Human Ethics Committee (Approval number HEC25032).

## Conflicts of Interest

The authors declare no conflicts of interest.

## Supporting information


**Supplementary File 1:** Interview schedule.

## Data Availability

Research data are not shared.
