# Supplementary material for: Staff Experiences Delivering Hospital Outreach Post‐Suicidal Engagement (HOPE) in a Regional Context: A Qualitative Study
Source: Int J Ment Health Nurs. 2026 Nov 10;35(6):e70401. doi: 10.1111/inm.70401 (PMC13749350; doi:10.1111/inm.70401)
Supplement: Supplementary file 1 [file INM-35-0-s001.docx]

Supplementary file 1: Interview schedule

**HOPE staff interview topic guide**

1. In your experience, what are the most common challenges faced when providing care for individuals with suicidal distress in the HOPE program?
2. How does the HOPE program currently collaborate with other agencies in the care of individuals with suicidal distress? (e.g. ED’s, police, paramedics, mental health services)
3. In your opinion, what factors or practices help improve care for individuals with suicidal distress in the HOPE program?
4. What changes or improvements would you suggest to develop a collaborative model of care for individuals experiencing suicidal distress in the HOPE program?
5. Are there any other ideas, strategies, or resources that you believe would help improve the care and support for people experiencing suicidal distress in the HOPE program?
